# Continuous enhancement of iturin A production by *Bacillus subtilis* with a stepwise two-stage glucose feeding strategy

**DOI:** 10.1186/s12896-015-0172-6

**Published:** 2015-06-09

**Authors:** Hu Jin, Kunpeng Li, Yanxing Niu, Mian Guo, Chuanjiong Hu, Shouwen Chen, Fenghong Huang

**Affiliations:** Oil Crops Research Institute, Chinese Academy of Agricultural Sciences, No. 2 Xudong Second Road, Wuhan, 430062 China; Hubei Key Laboratory of Lipid Chemistry and Nutrition, Wuhan, 430062 China

**Keywords:** *Bacillus subtilis*, Iturin A, Lipopeptide, Rapeseed meal, Fed-batch fermentation

## Abstract

**Background:**

The lipopeptide antibiotic iturin A is an attractive biopesticide with the potential to replace chemical-based pesticides for controlling plant pathogens. However, its industrial fermentation has not been realized due to the high production costs and low product concentrations. This study aims to enhance iturin A production by performing a novel fermentation process with effective glucose feeding control using rapeseed meal as a low-cost nitrogen source.

**Results:**

We demonstrated that continuous and significant enhancement of iturin A production could be achieved by a novel two-stage glucose-feeding strategy with a stepwise decrease in feeding rate. Using this strategy, the ratio of spores to total cells could be maintained at a desirable/stable level of 0.80–0.86, and the reducing sugar concentration could be controlled at a low level of 2–3 g/L so that optimal substrate balance could be maintained throughout the feeding phase. As a result, the maximum iturin A concentration reached 1.12 g/L, which was two-fold higher than that of batch culture.

**Conclusions:**

This is the first report which uses control of the glucose supply to improve iturin A production by fed-batch fermentation and identifies some important factors necessary to realize industrial iturin A production. This approach may also enhance the production of other useful secondary metabolites by *Bacillus subtilis.*

## Background

Concerns regarding a healthy food supply and pesticide resistance in commercial crops have encouraged the development of biological control methods to replace the extensive use of chemical-based pesticides, thereby achieving safer and more effective pest and disease control [1, 2]. Biological control is the use of natural antagonistic organisms to combat pests or suppress plant diseases [3]. *Bacillus subtilis*, one of the most commonly used and well-studied microbial species, has the potential to produce more than two dozens of structurally diverse broad spectrum antimicrobial compounds with high viability [4]. Among these antimicrobial compounds, cyclic lipopeptides of the iturin, surfactin and fengycin families have well-recognized potential uses in biotechnology and biopharmaceutical applications because of their excellent surfactant properties.

Iturin A is a cyclic lipopeptide antibiotic consisting of a cyclic heptapeptide linked to a 14–17 carbons β-amino fatty-acid chain [5]. This special amphipathic structure endows iturin A with strong broad-spectrum antifungal activity so that it could be used as a potential bio-control agent against harmful plant pathogens that cause crop diseases [6, 7]. Lipopeptide antibiotics have great commercial, therapeutic and environmental application potentials. However, the production of lipopeptide antibiotics including iturin A on an industrial scale has not been realized, due to the high production costs and low product yields [8]. In general, the raw material costs account for 30–40 % of the total production costs in most biotechnological processes [9]. Hence using low cost raw materials from abundant sources may be an important aspect to improve the economic viability of industrial lipopeptide production. Recently, a wide variety of those raw materials including agro-based byproducts [10–12] and industrial wastes [13–15] have been used as substrates for iturin A or surfactant production. In addition to reducing production costs, many studies have also been carried out to improve product yields/concentrations by optimizing cultivation conditions or screening for overproducing mutants or creating recombinant strains [16, 17]. An efficient bioprocess with low production costs and high yield is extremely important for cost-effective commercial production of lipopeptide antibiotics [9].

Secondary metabolites including iturin A are generally produced after the logarithmic cell growth phase when one or more essential nutrients become deficient [10]. In addition, lipopeptide antibiotics synthesis is regulated by mechanisms associated with starvation-induced systems such as sporulation [18]. Sporulation of *B. subtilis* is a natural phenomenon which occurs in response to starvation, however the complete transformation of metabolically active cells to spores will eventually terminate the production of lipopeptides [19]. It has been suggested that the second stage production of iturin A could be induced by the germination of spores through heat-activation and nutrient supplementation [19]. Thus, the nutrient supply is necessary for reproduction of iturin A by activating/recovering spores into metabolically active cells. The nutrient supply should be strictly controlled, as excessive nutrients in the culture broth would lead to a high *B. subtilis* growth but cessation of iturin A production [10]. Because of the complex correlations between iturin A production and the sporulation/nutrient requirement characteristics of *B. subtilis*, it is critical that a limited nutrient supply should be used in order to recover the optimal number of metabolically active cells. To date, improving iturin A production by manipulation of the nutrient supply in fed-batch fermentation has seldom been reported.

In our previous study, the feasibility and effectiveness of directly utilizing rapeseed meal as an easily obtainable, low cost, nitrogen-rich substrate for iturin A production, were testified in submerged batch fermentation [20]. In the present study, we attempted to further improve the iturin A yield by effectively supplementing glucose in fed-batch fermentation. Firstly, we determined the optimal starting feeding time in flask fermentations with pulsed substrate feeding. Subsequently, the characteristics of iturin A production, glucose consumption and spores formation with different glucose feeding rates in fed-batch fermentations were investigated and evaluated in a bioreactor. Finally, a novel two-stage stepwise decreased glucose feeding strategy was proposed for efficient iturin A production.

## Results and discussion

### Iturin A production by *Bacillus subtilis* in shake flask batch cultivation

**Fig. 1:**
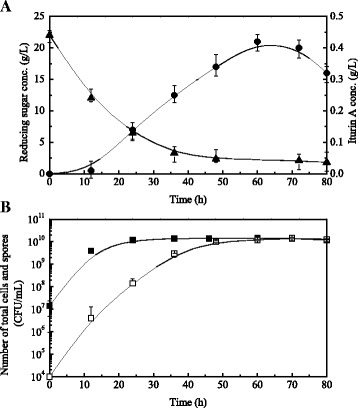
Time courses of reducing sugar and iturin A concentrations (**a**) and number of total cells and number of spores (**b**) using rapeseed meal as nitrogen sources in flask batch fermentation. Symbols: iturin A concentration (●), reducing sugar concentration (▲), number of total cells (■), number of spores (□). Each point represents the mean (*n* = 3) ± standard deviation

Iturin A production, reducing sugar concentration, and number of total cells (including spores and vegetative cells) during batch fermentation in flasks are shown in Fig. 1. During the first 24 h, the reducing sugar concentration decreased rapidly from 22.0 g/L (initial) to 6.4 g/L (Fig. 1a). Over the same time period, cells grew exponentially and the number of total cells increased from the initial 1.2 × 10^7^ to 1.4 × 10^10^ CFU/mL (Fig. 1b). After 24 h, the reducing sugar concentration decreased slowly and reached a stable level of about 2 g/L till the end of fermentation. As shown in Fig. 1a, the production of iturin A increased gradually after 12 h, and a maximum iturin A concentration of 0.42 g/L was reached at 60 h. After 24 h, during the stationary phase, the total cell number remained unchanged, but the number of spores increased gradually with culture time and reached a maximum level at approximately 72 h (Fig. 1b). As shown in Fig. 1, almost of all the vegetative cells became spores when reducing sugar was deficient, and iturin A production stopped correspondingly.

### Fed-batch fermentation by pulsed substrate feeding in flasks

The production of secondary metabolites such as iturin A is closely related to the nutrient supply. Iturin A was largely produced after the exponential growth phase when nutrients were near deficient [10]. However, the complete depletion of one or more essential nutrients usually results in spores formation from metabolically active vegetative cells and eventually iturin A production ceases [19]. Fig. 1 demonstrates that iturin A production ceased and then began to decrease when the metabolically active vegetative cells were completely transformed into spores under substrate exhaustion conditions. Moreover, the reducing sugar concentration remained almost constant after 35 h.

**Fig. 2:**
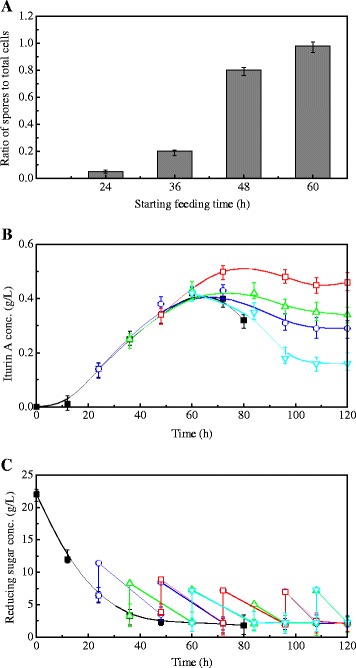
The relationship of spores to total cells ratios and starting feeding time (**a**), time courses of iturin A concentration (**b**) and reducing sugar concentration (**c**) in flasks using a pulsed glucose-feeding strategy with different feeding initiating times. Symbols: control (■), feeding at 24 h (○), feeding at 36 h (△), feeding at 48 h (□), feeding at 60 h (▽). Each point represents the mean (*n* = 3) ± standard deviation

To overcome the above problem and extend the iturin A production period, pulsed addition of reducing sugar was considered and the optimal initial feeding conditions were explored. To avoid the inhibitory effect of high glucose concentration on iturin A production, 0.3 mL glucose feeding medium was fed into 30 mL broth every 24 h (an average glucose feeding rate of 0.21 g/L/h) after initiating glucose supplementation, until the end of fermentation. Figure 2 shows the results of initiating substrate feeding at various time points, 24, 36, 48 and 60 h, during the stationary growth phase in flask experiments. The corresponding spores to total cells ratios were 0.05, 0.20, 0.80 and 0.98 respectively (Fig. 2a). As shown in Fig. 2b, iturin A production could be continuously increased by the pulsed addition of glucose, and the optimal feeding initiating time was 48 h, when the spores to total cells ratio was 0.80, (Fig. 2a). In this case, the maximum iturin A concentration was 0.5 g/L at 72 h, which was 19 % higher than that of control (no glucose feeding). Feeding at a later stage in the stationary phase (60 h, spores to total cells ratio close to 1, Fig. 2a) did not further improve iturin A production (Fig. 2b). The pulsed feeding strategy did not cause glucose accumulation at any feeding time point, and the final glucose concentrations in all runs were at similar low levels (Fig. 2c), indicating that the glucose added during each feeding cycle (a feeding rate of 0.21 g/L/h) could be consumed completely.

### Fed-batch fermentation with constant glucose-feeding rates in a 7-L bioreactor

**Fig. 3:**
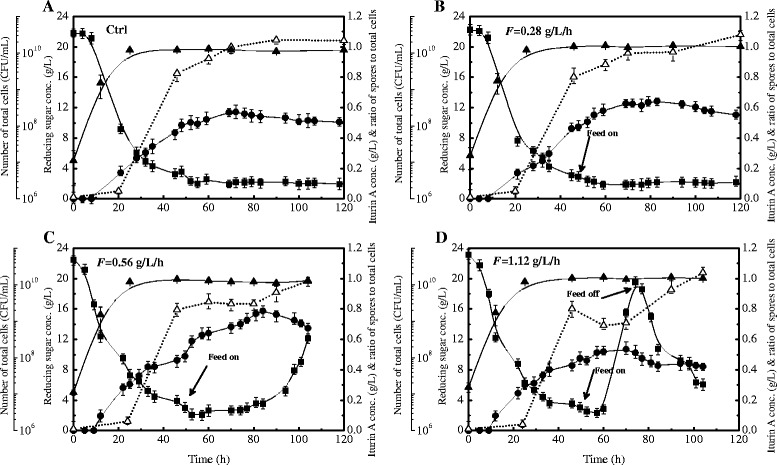
Key fermentation parameters with different glucose-feeding rates. The garph shows the changing patterns of total cells numbers, reducing sugar concentrations, iturin A concentrations and spores to total cells ratios in four fermentation runs with different glucose-feeding rates. Ctrl, F=0 g/L/h (**a**), F=0.28 g/L/h (**b**), F=0.56 g/L/h (**c**) and F=1.12 g/L/h (**d**). Symbols: iturin A concentration (●), reducing sugar concentration (■), number of total cells (▲), ratio of spores to total cells (△). Each point represents the mean (*n* = 3) ± standard deviation

To investigate the effect of different glucose-feeding rates on iturin A production, we scaled up and conducted the fermentations in a 7-L bioreactor, where glucose-feeding rates, pH, and dissolved oxygen (DO) concentration could be easily controlled or monitored. Figure 3 depicts the total cells number, reducing sugar and iturin A concentrations, and spores to total cells ratios in four fermentation runs with different glucose-feeding rates. In all of the fermentation runs, the total cells numbers increased rapidly during the first 24 h and then remained unchanged. The spores numbers also increased in accordance with the increase in total cell numbers and the spores to total cells ratio approached 1 at approximately 70 h, indicating that almost all of the metabolically active vegetative cells became spores at this time. During the exponential growth phase (0–24 h), the reducing sugar concentration decreased rapidly (Fig. 3a). However, the reducing sugar did not decline further after 50 h and remained at a low level of 2 g/L until the end of fermentation. This fact suggested that some unknown but unusable sugar was released from rapeseed meal. The maximum iturin A concentration in bioreactor-scaled batch fermentation (no glucose feeding) reached 0.57 g/L at 72 h (Fig. 3 a), which was 35.7 % higher than that in flask-based batch fermentation (Fig. 1a).

In the flask-based experiments, iturin A production could be enhanced continuously and the iturin A production period could be prolonged effectively by pulsed glucose feeding (Fig. 2). In this case, all of the glucose added was completely consumed, but the sudden change in glucose concentration during the pulsed glucose feeding could deteriorate iturin A production. To eliminate the large environmental changes due to the pulsed glucose addition and to further improve iturin A production, a constant glucose feeding strategy was considered and fermentations with three different constant glucose feeding rates were conducted. Based on the results of the flask experiments, glucose constant feeding was initiated at 48 h for the three batches.

As shown in Fig. 3b, when the glucose feeding rate was controlled at a low level of 0.28 g/L/h, the added glucose was quickly consumed and no reducing sugar accumulation was observed throughout the feeding period. With this feeding rate, the maximum iturin A concentration reached 0.62 g/L, an increase of 12.3 % compared with batch fermentation (0.57 g/L). On the other hand, spores formation could not be reduced and a continuous increase in the spores to total cells ratio could not be controlled with this low glucose feeding rate (Fig. 3b). A significant increase in iturin A production could be achieved if the glucose feeding rate was raised to a moderate level of 0.56 g/L/h, as shown in Fig. 3c. In this case, the maximum iturin A concentration of 0.78 g/L was obtained at 82 h, which was 36.8 % higher than that of the batch run. Furthermore, with this feeding rate, the spores to total cells ratio could at least be maintained at about 0.80 for a period of 30 h. This observation indicates that the ability of *B. subtilis* to utilize glucose changed throughout the feeding period (Fig. 3). During the early stage (50–80 h), reducing sugar concentration could be maintained at a stable and lower level of 3–4 g/L, indicating that *B. subtilis* had an elevated ability to utilize glucose. After 80 h, the ability of cells to utilize glucose gradually reduced, leading to a continuous increase in reducing sugar concentration and severe reducing sugar accumulation of up to 12 g/L at the end of fermentation. As a result, the concentration of iturin A decreased correspondingly, and the spores to total cells ratio began to rise and was out of control (Fig. 3c). The fermentation performance was examined again when the glucose feeding rate was raised to a further higher level (1.12 g/L/h). In this case, the spores to total cells ratio quickly decreased from 0.8 to 0.7 and then remained at this level for the first 10 h after feeding, suggesting that high glucose feeding rate activated the germination of spores to metabolically active vegetative cells. However, the rapid glucose utilization period (50–60 h) could only be sustained for a short time. Glucose then began to accumulate quickly to a level of up to 20 g/L at 76 h (Fig. 3d). Glucose accumulation resulted in reduced iturin A production and a gradual rise in spores to total cells ratio. The highest iturin A concentration remained at a low level of 0.5 g/L.

### Changing patterns of dissolved oxygen and pH in batch and fed-batch fermentations with different feeding rates

**Fig. 4:**
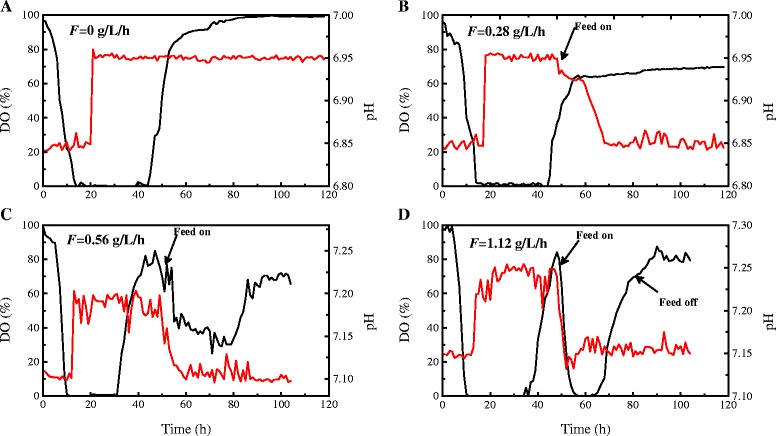
Time courses of pH and DO with different glucose feeding rates. Graphs show a time course of pH and DO and its correlation with different glucose feeding rates during fermentation. F=0 g/L/h (**a**), F=0.28 g/L/h (**b**), F=0.56 g/L/h (**c**) and F=1.12 g/L/h (**d**). Solid black lines: DO; solid red lines: pH

Dissolved oxygen (DO) and pH are two conventional state variables used for monitoring the fermentation process, and to some extent, their changing patterns reflect metabolic characteristics of the cultured cells and provide some useful information for industrial process control. The on-line changing patterns of pH and DO in batch and fed-batch fermentations with different feeding rates are shown in Fig. 4. In batch fermentation using rapeseed meal as a nitrogen source, DO decreased to 0 % rapidly during the early stage (0–10 h) and then remained at 0 % until 42 h (Fig. 4a), indicating a high oxygen demand during this period. DO began to rise gradually after 42 h when depletion of fermentable sugars occurred (Fig. 3a). On the other hand, pH remained at its low limit during the early stage (0–20 h), then it rose continuously and was maintained at its high limit by continuous addition of acid solution till the end of fermentation (Fig. 4a). The sudden rise in pH during the exponential growth phase suggested that rapeseed meal protein had to be utilized as an alternative carbon source to meet the high carbon demand for rapid cell growth. However, in contrast with batch fermentation, the pH began to decrease gradually and could be maintained at its low limit by addition of alkaline solution at all glucose feeding conditions (Fig. 4). On the other hand, the DO changing patterns varied with glucose-feeding rates. A lower glucose feeding rate (0.28 g/L/h) strategy could not control the continuous increase in DO and the pattern of change was similar to that of batch fermentation (Fig. 4a and b). This implies that cellular metabolic activity could not be improved by a low rate of glucose-feeding, which also coincided with the fact that the spores to total cells ratio remained unchanged compared with batch fermentation (Fig. 3b). When adopting the moderate glucose-feeding rate (0.56 g/L/h) strategy, DO significantly decreased after initiating glucose-feeding and then was maintained at 30–40 % for a period of 30 h. At 80 h, DO began to rise gradually again when reducing sugar accumulated (Figs. 4c and Fig. 3c). Consistently, the higher glucose feeding rate strategy resulted in a rapid DO decrease during the first 10 h after initiating glucose feeding. However, DO rapidly rebounded at about 60 h when glucose accumulation occurred due to over-feeding (Figs. 3d and 4d).

### Enhanced iturin A production with a two-stage glucose feeding strategy by a stepwise decrease in feeding rate

**Fig. 5:**
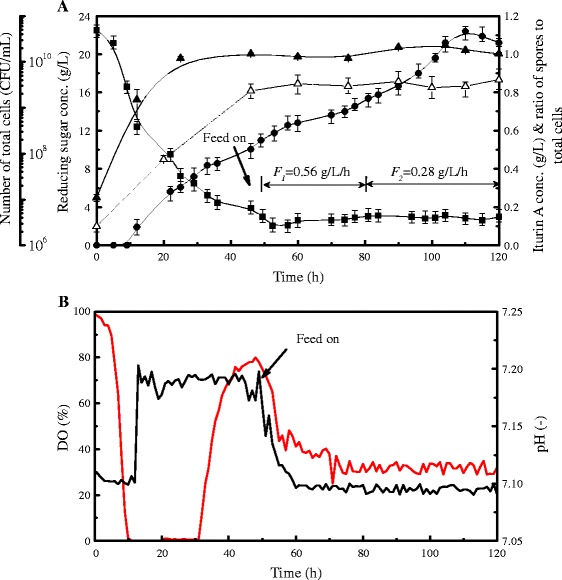
Time courses of fed-batch fermentation performance with the two-stage glucose-feeding strategy by stepwise decrease in feeding rate in a 7-L bioreactor. Graphs show variation in key parameters including number of total cells, reducing sugar concentration, iturin A concentration and ratio of spores to total cells (**a**) as well as DO and pH (**b**) during the fermentation over time. Symbols: iturin A concentration (●), reducing sugar concentration (■), number of total cells (▲), spores to total cells ratio (△). Each point represents the mean (*n* = 3) ± standard deviation. Solid black lines: DO; solid red lines: pH

The fed-batch fermentation results in the bioreactor indicated that a lower glucose feeding rate of 0.28 g/L/h was not enough to induce the generation of spores, and the maximum iturin A concentration only increased 12.3 % compared with that of the batch fermentation (Fig. 3b). In contrast, a high glucose feeding rate of 1.12 g/L/h resulted in severe glucose accumulation and reduced iturin A production (Fig. 3d). Although the moderate glucose feeding rate of 0.56 g/L could significantly enhance iturin A production, reducing sugar accumulation still occurred at 80 h fermentation due to the reduced glucose consumption rate during late fermentation (Fig. 3c). As the glucose consumption rate varied during the entire feeding period, thus, a novel two-stage glucose feeding strategy was proposed, in which glucose feeding rate was controlled at a moderate level of 0.56 g/L/h during the early feeding stage (48–80 h) and then decreased to a low level of 0.28 g/L/h during the late feeding stage (80–120 h). The aim of the proposed strategy was to maintain an optimum glucose concentration for iturin A production. As shown in Fig. 5, with this two-stage glucose feeding strategy, reducing sugar concentration could be controlled at a low level of 2–3 g/L throughout the feeding period. At the same time, the spores to total cells ratio also was stable at about 0.80-0.86 throughout the feeding period. After initiating the feeding at 48 h, DO fell gradually and then remained at a constant level of 30 % until the end of fermentation, reflecting the maintenance of a relatively stable and high cellular metabolic activity under this condition. With the proposed feeding strategy, iturin A production increased continuously reaching a maximum of 1.12 g/L at 110 h, which was two-fold higher than that of batch fermentation.

### Fermentation performance comparison using different feeding strategies

The major performance indices observed during different feeding strategies are summarized in Table 1. Compared to batch fermentation, an effective supply of glucose was beneficial for continuous increase of iturin A production. Notably, maintaining the spores to total cells ratio at a suitable level (about 0.80) was critical for improving iturin A fermentation performance, and the desired ratio could be achieved by adaptively adjusting the glucose feeding rate to suit the changing cellular metabolic characteristics during the feeding phase (Table 1). As shown in Table 1, the maximum total cell numbers for both batch or fed-batch fermentation were almost the same, indicating that glucose feeding only influenced the germination of spores rather than altering the growth of total cells. A low glucose feeding rate was not sufficient for effective germination of spores, so that iturin A production could not be enhanced. A high glucose feeding rate (1.12 g/L/h) resulted in rapid glucose accumulation (Fig. 3d) and huge fluctuations in the spores to total cells ratio, which led to a deterioration in iturin A production. Both iturin A concentration and productivity could be maintained at higher levels when controlling the glucose feeding rate at a moderate level of 0.56 g/L/h. The highest iturin A concentration of 1.12 g/L was obtained by adopting the proposed two-phase feeding strategy by a stepwise decrease in the feeding rate. With the proposed two-phase feeding strategy, the iturin A production period was prolonged from 72 h to 110 h, and the spores to total cells ratio could be maintained at a desirable/stable level of 0.80–0.86 throughout the feeding phase.

**Table 1:** Comparison of major fermentation performance index with different fermentation strategies

Fermentation strategy	Iturin A concentration at 72 h (g/L)	Maximum Iturin A conc. (g/L) & time	Maximum total cell number (CFU/mL)	Ratio of spores to total cells after 48 h	Iturin A productivity at 72 h (g/L/h)
Batch fermentation	0.57	0.57 (72 h)	1.3 × 10^10^	0.82–1.04	0.0079
Low feeding rate (*F* = 0.28 g/L/h)	0.62	0.64 (83 h)	1.7 × 10^10^	0.80–1.08	0.0086
Moderate feeding rate (*F* = 0.56 g/L/h)	0.70	0.78 (84 h)	1.4 × 10^10^	0.79–0.92	0.0097
High feeding rate (*F* = 1.12 g/L/h)	0.51	0.53 (70 h)	1.6 × 10^10^	0.69–1.04	0.0071
Two-stage feeding strategy (*F* = 0.56 → *F* = 0.28 g/L/h)	0.68	1.12 (110 h)	1.4 × 10^10^	0.80–0.86	0.0094

For industrial-scale iturin A production, the optimization of initial fermentation conditions and medium components in flasks are usually the first and necessary steps once an iturin A over-producing *B. subtilis* strain has been selected. Response surface methodology (RSM) has been commonly used as a tool to identify optimal fermentation conditions for iturin A production [21, 22]. However, after obtaining the optimal initial fermentation conditions (including medium components and environmental conditions), effective control of the fermentation process to realize a high and stable yield in bioreactor is essential. From the point of view of process control, our current study focused on further improving iturin A production by altering glucose feeding based on the iturin A production, cell growth and glucose consumption characteristics. Our investigation proved the effectiveness of a two-stage glucose feeding strategy for continuous improvement of iturin A production.

## Conclusions

In the present study, a two-stage glucose-feeding strategy by stepwise decrease in feeding rate was proposed to adaptively respond to variation in glucose consumption rate throughout the feeding phase. With the proposed feeding strategy, the spores to total cells ratio could be maintained at the desirable/stable level of 0.80–0.86, glucose deficiency and over-accumulation could be avoided simultaneously. As a result, the highest iturin A concentration reached 1.12 g/L, which was two-fold higher than that of batch culture. The proposed control strategy could also have future potential application in enhancing the production of other secondary metabolites by *Bacillus subtilis.*

## Methods

### Microorganism

The iturin A production strain of *Bacillus subtilis* 3–10 (GeneBank accession number JF460845), was isolated from a soil sample collected from a field in a suburb of Wuhan.

### Medium

The LB medium used for seed culture had the following composition (in g/L, unless otherwise specified): tryptone 10, yeast extract 5, NaCl 10. In addition, 20 g/L agar was added to the slant medium. The fermentation medium was composed of (in g/L) glucose 20, K_2_HPO_4_•3H_2_O 1, MgSO_4_•7H_2_O 0.5, MnSO_4_•H_2_O 0.005, and rapeseed meal 90. The initial pH of the medium was adjusted to 7.0 and autoclaved at 121 °C for 30 min. The feeding medium for fed-batch experiments was composed of 500 g/L glucose.

### Fermentation conditions

For seed preparation, *Bacillus subtilis* 3–10 from a fresh slant was inoculated into 30 mL seed medium in 250 mL flasks and cultivated in a rotary shaker at 220 rpm for 12 h. Bioreactor experiments were performed in a 7 L bench-scaled bioreactor (BIOSTAT® A Plus, Sartorius Stedim Biotech, Germany) with the initial working volume of 3 L. The inoculation size was 2 % (v/v). The feeding speeds of glucose for fed-batch cultures were controlled via a speed-adjustable peristaltic pump (Longer Pump Co., China). All fermentations were carried out at 28 °C and pH was maintained automatically at 7.0 by the addition of 4 M NaOH or 4 M H_2_SO_4_ solutions. Aeration and agitation rates were controlled at 2 vvm and 600 rpm, respectively. During the fermentations, samples were taken at 4–12 h intervals for off-line analysis.

### Determination of total cells and spores number

The number of total cells (including active vegetative cells and spores) during submerged fermentation was determined as follows: 0.5 mL of sample was taken into a sterile 10 mL test tube, and mixed with 4.5 mL of sterile distilled water and shaken at 150 rpm using a vortex for 5 min at room temperature. Then, the mixture was serially diluted and spread onto LB-agar plates. After 24 h of incubation at 28 °C, the number of colonies was counted and expressed as colony forming units (CFU). For determination of the spores number, the above serially diluted mixture was further heat-treated at 80 °C for 15 min to kill the vegetative cells in the diluted sample and then the same procedure for colony counting was applied [19].

### Extraction and quantitation of iturin A

Iturin A was extracted according to the reported method [10] with some modifications: 300 μL of strain 3–10 culture was suspended in a microtube containing 1200 μL methanol and then the mixture was shaken at room temperature for 60 min. The mixture was centrifuged at 12,000 rpm for 20 min, and the supernatant was filtered through a 0.22-μm pore-size hydrophobic polytetrafluoroethylene (PTFE) syringe filter unit. The iturin A concentration in the filtrate was quantified with a Waters 2695 HPLC system equipped with a reverse-phase HPLC column (ACQUITY UPLC BEA C18 1.7 μm 2.1 × 100 mm, Waters, USA) at a flow rate of 0.3 mL/min. A mixture of acetonitrile and 10 mM ammonium acetate (35:65, v/v) was used as the eluent and the elution was monitored at 210 nm. Iturin A standard (Sigma Chemicals, St. Louis, MO) was used to determine the calibration line. The contents of iturin A and measurement deviation at different sampling times were determined using triplicate samples.

### Measurement of reducing sugar concentrations

The fermentation samples were centrifuged at 12,000 rpm for 20 min, and the supernatant was used for measuring reducing sugar and soluble protein concentrations. The reducing sugar concentrations were determined by the DNS method using 3, 5-dinitrosalicylic acid reagent [23].
